# Post-stimulus beta responses are modulated by task duration

**DOI:** 10.1016/j.neuroimage.2019.116288

**Published:** 2020-02-01

**Authors:** Daisie O. Pakenham, Andrew J. Quinn, Adam Fry, Susan T. Francis, Mark W. Woolrich, Matthew J. Brookes, Karen J. Mullinger

**Affiliations:** a Sir Peter Mansfield Imaging Centre, School of Physics and Astronomy, University of Nottingham, Nottingham, NG7 2RD, UK; b Oxford Centre for Human Brain Activity, Wellcome Centre for Integrative Neuroimaging, Department of Psychiatry, University of Oxford, UK; c Department of Rehabilitation Medicine, Icahn School of Medicine at Mount Sinai, New York, NY, USA; d Centre for Human Brain Health, School of Psychology, University of Birmingham, UK

## Abstract

Modulation of beta-band neural oscillations during and following movement is a robust marker of brain function. In particular, the post-movement beta rebound (PMBR), which occurs on movement cessation, has been related to inhibition and connectivity in the healthy brain, and is perturbed in disease. However, to realise the potential of the PMBR as a biomarker, its modulation by task parameters must be characterised and its functional role determined. Here, we used MEG to image brain electrophysiology during and after a grip-force task, with the aim to characterise how task duration, in the form of an isometric contraction, modulates beta responses. Fourteen participants exerted a 30% maximum voluntary grip-force for 2, 5 and 10 s. Our results showed that the amplitude of the PMBR is modulated by task duration, with increasing duration significantly reducing PMBR amplitude and increasing its time-to-peak. No variation in the amplitude of the movement related beta decrease (MRBD) with task duration was observed. To gain insight into what may underlie these trial-averaged results, we used a Hidden Markov Model to identify the individual trial dynamics of a brain network encompassing bilateral sensorimotor areas. The rapidly evolving dynamics of this network demonstrated similar variation with task parameters to the ‘classical’ rebound, and we show that the modulation of the PMBR can be well-described in terms of increased frequency of beta events on a millisecond timescale rather than modulation of beta amplitude during this time period. Our results add to the emerging picture that, in the case of a carefully controlled paradigm, beta modulation can be systematically controlled by task parameters and such control can reveal new information as to the processes that generate the average beta timecourse. These findings will support design of clinically relevant paradigms and analysis pipelines in future use of the PMBR as a marker of neuropathology.

## Highlights

• The post-movement beta rebound is modulated by task duration.
• Increasing task duration reduces amplitude of the post-movement beta rebound.
• The modulation is explained by increased frequency of short-timescale beta events.

## 1 Introduction

Motor tasks typically generate electrophysiological responses in the beta (15–30 Hz) frequency band (Jurkiewicz et al., 2006). Such responses comprise a decrease in amplitude during movement - the movement related beta decrease (MRBD) - followed by an increase in amplitude above baseline on movement cessation - the post-movement beta rebound (PMBR). These phenomena are well documented (Cheyne, 2013; Kilavik et al., 2013; Pfurtscheller & Lopes da Silva, 1999), yet a full understanding of how they are modulated by stimulus parameters and their functional roles remains unknown.

The MRBD is not only observed during movement but also during motor planning (Tzagarakis et al., 2010) and imagining movements (Pfurtscheller et al., 2005; Schnitzler et al., 1997) (albeit at lower amplitude). Previous work has shown that the MRBD amplitude, duration and onset time is modulated by task parameters such as certainty of movement or number of movement options. For example, Tzagarakis and colleagues (Tzagarakis et al., 2010) showed that during movement preparation (i.e. prior to actual movement onset), the drop in beta oscillatory amplitude was significantly greater in a case where the direction of movement was certain, than a case where the direction of movement was uncertain. However, during movement itself, the MRBD has been shown to be relatively unaffected by parameters such as force output, rate of force development (Fry et al., 2016), or speed of force development (Stancak and Pfurtscheller, 1995, 1996). This has led to a hypothesis that the MRBD relates to movement planning and execution, but not to measurable changes in peripheral output.

The PMBR has also been shown to be modulated by a number of task parameters. Stevenson et al. (2011) measured MEG responses to finger abductions performed for a range of durations (1, 2, 4 and 6 s) and found an increase in the total PMBR with increased task duration, which plateaued after stimulus durations of 4 s. Another study (Parkes et al., 2006) showed that the rate of finger extensions affects PMBR, with faster movements resulting in a higher amplitude. The PMBR has been found to be larger for incorrect compared to correct button presses (Koelewijn et al., 2008). Heinrichs-Graham et al. (2017) showed the PMBR is stronger for cues to terminate movement at 2 s compared to 2.5 s. It is therefore evident that the PMBR can be modulated by movement parameters. A number of studies have also shown modulation of PMBR across subjects; for example, Gaetz et al. (2010) found that the PMBR is significantly reduced in children and diminished in adolescents compared to adults. Vakhtin et al. (2015) showed similar findings and suggested that the PMBR is modulated by age in a predictable manner in adolescents. Perhaps most importantly, the PMBR is modulated by disease, opening the potential for its use as a biomarker. For example, Robson et al. (2016) showed that patients with schizophrenia have a smaller PMBR compared to healthy controls, and the amplitude of the response decreases with increasing symptom severity. In a study of autism, the PMBR was found to be reduced when patients were observing hand movements compared with healthy controls (Honaga et al., 2010). In a study of stroke patients, Parkkonen et al. (2017) found the PMBR was decreased bilaterally (i.e. independent of affected side) in patients during passive finger movements compared with controls. Barratt et al. (2017) found patients with multiple sclerosis had delayed PMBR compared to healthy controls. Proudfoot et al. (2017) showed a delayed PMBR and larger MRBD during movement execution in patients with amyotrophic lateral sclerosis. These results suggest that the PMBR is functionally important, and the generation of a better understanding of its role may lead to its use as an effective biomarker across a number of disorders.

The fact that the MRBD and PMBR differ in their response to stimulus parameters, individual differences and disease suggests that they also have different neuronal generators (Parkkonen et al., 2015). This is supported by a number of studies showing that the generator of the PMBR is anterior in the brain compared to the MRBD (Fry et al., 2016; Jurkiewicz et al., 2006; Salmelin et al., 1995; Stancak and Pfurtscheller, 1995). The MRBD has been described as a “cortical gate” to facilitate local processing in sensory and motor cortex (Fry et al., 2016; Stevenson et al., 2011), whereas the PMBR might provide active inhibition of motor cortex (Cassim et al., 2001; Stevenson et al., 2011). This latter hypothesis is supported by a measurable relationship between the concentration of gamma-aminobutyric acid (GABA) and the PMBR (Cheng et al., 2017; Gaetz et al., 2011; Hall et al., 2011; Muthukumaraswamy et al., 2013). It has further been suggested that whilst the MRBD might represent local processing, the PMBR is likely to relate to long range integrative processes over distributed networks (Tewarie et al., 2018). This also agrees with resting state studies showing that long range networks are mediated by beta band oscillations (Hipp et al., 2012). It is therefore tempting to suggest that the PMBR is representative of top-down inhibitory control of the primary motor region, by a wider sensorimotor and premotor network.

A recent body of work describes beta oscillations in terms of a “bursting” hypothesis (Little et al., 2018; Sherman et al., 2016). The premise is that, distinct from the view of an ongoing oscillation whose amplitude changes over time, beta “oscillations” are generated by short punctate events, or bursts, that are not necessarily time-locked over trials. The MRBD can be thought of as an absence of bursts, whilst the PMBR reflects an increased burst likelihood which, when averaged over trials, looks like a smooth increase in oscillatory amplitude (Little et al., 2018). The idea that electrophysiological data can be broken down into punctate events is not new, indeed it has been supported by a vast body of evidence that decomposes whole brain electrophysiological data, measured using EEG, into “microstates” (Koenig et al., 2005; Lehmann et al., 1998) that represent short (~100-ms) windows, in which the distribution of EEG power over the scalp remains stable. More recently, studies (Baker et al., 2014; Vidaurre et al., 2018a, Vidaurre et al., 2018b) have used a Hidden Markov Model (HMM) to identify points in time at which distinct spatial patterns of oscillatory power occur. Results show that brain activity can be parcellated into ‘states’, each of which has a spatial signature that relates to canonical resting state networks. These networks, including the sensorimotor network, modulate on a very short (100 ms) time scale. Novel methods, like the HMM, potentially offer a new means to understand the nature of the MRBD and the PMBR, and their perturbation in disease in the context of beta bursts and network dynamics. However, this field is still unfolding (van Ede et al., 2018), and to date the relationships between bursts, connectivity, and classical metrics like the PMBR remain unclear.

Ultimately, to realise the potential of the PMBR as a biomarker of disease, it is critical that it is consistently and correctly characterised, and a better understanding of its functional role is developed. Understanding how the PMBR is affected by task parameters is important to reduce variance within cohorts and better disassociate disease types from variation due to the way a task is performed. However, the characterisation of PMBR variation with task parameters remains poorly documented. For example, precise movement parameters (i.e. rate, force of movement etc.) are rarely recorded and although Pfurtscheller et al. argued in 1999 that it was necessary to leave 10 s between movements to allow the PMBR to return to a true baseline (Pfurtscheller & Lopes da Silva, 1999), this has rarely been adhered to. These arguments show the increasing importance of developing a new generation of well controlled motor tasks with long inter-stimulus intervals for use in electrophysiology investigations. In terms of characterising the PMBR, the changing literature on beta bursts and network connectivity, coupled with novel methodologies like the HMM, offer new opportunities to process and ultimately understand the evolution of the PMBR in the context of brain network architecture. Here, we seek to bring these areas together, using a well-controlled motor task to fully parameterise the changes in beta band oscillations with task duration, and combining conventional analysis and novel network modelling (HMM) to interrogate the relationship between brain states, network dynamics and the PMBR.

## 2 Methods

### 2.1 Subjects

Fifteen healthy volunteers (10 female, aged 27 ± 3 (mean ± SD) years) took part in this study, which was approved by the University of Nottingham Medical School Research Ethics Committee. All volunteers gave written, informed consent and self-reported as being right-handed.

### 2.2 Motor paradigm

Maximum voluntary force (MVF) was determined for each individual subject prior to the start of the experiment. Subjects were encouraged to exert their maximum force using a grip-force bar (Current Designs, Philadelphia, USA) for a period of 1–2 s, with 2 repeats separated by ~15 s. The MVF was taken as the peak maximum force averaged over a 200-ms epoch achieved in either repeat, compared with the baseline reading of the force bar (mean over 400 ms at the end of the recording). A target force for the MEG study was then set at 30% of the subject’s MVF.

**Fig. 1:**
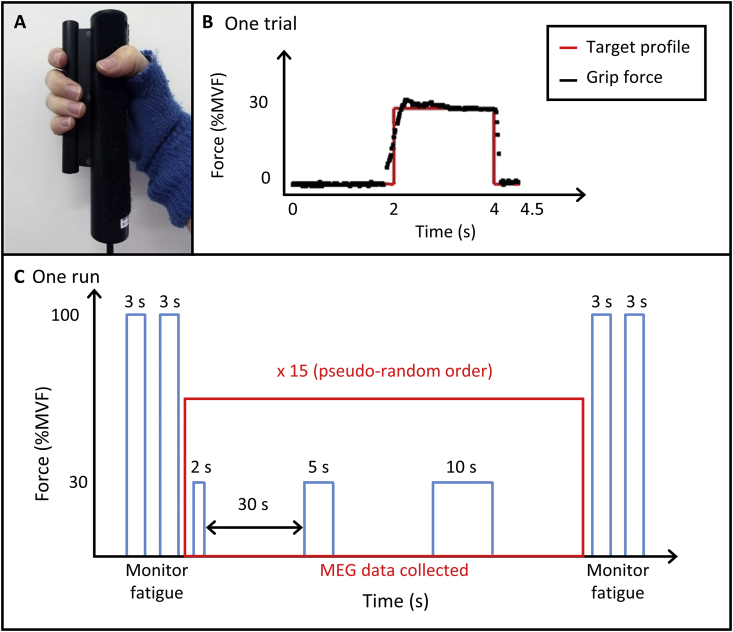
**Overview of the experiment.** (A) The grip-force bar (Current Designs, Philadelphia, USA) attached to a fingerless glove to allow relaxation of the hand. (B) Example single trial. The target force profile is shown (red) with real-time force output from a single trial overlaid (black). The visual stimulus appeared 2 s before the force output period, which was sustained for 2, 5 or 10 s [shown here for 2 s]. The profile remained on the screen for a further 0.5 s after the end of the force output period and was followed by a fixation cross for 27.5 s. (C) Schematic diagram of one run. Single trials were repeated 15 times for each duration in a pseudo-random order, totalling 45 trials within one run. This was followed by a second run after an approximately 15-min break. Two 3-s target profiles of 100%MVF were presented before and after each run to monitor fatigue.

Subjects lay supine with their head resting in the MEG helmet and held a grip-force bar in their right hand (Fig. 1A). Subjects applied a force to the bar when visually cued. The visual stimulus comprised a target profile of the required force output, which appeared 2 s before the stimulus period onset. During the stimulus period, subjects were instructed to squeeze the grip-force bar to match the target profile at 30%MVF for periods of either 2, 5 or 10 s. The force output was measured directly and overlaid onto the target profile in real-time, to provide visual feedback (see Fig. 1B). The target profile remained on the screen 0.5 s after the end of the stimulus. A fixation cross was then presented on the centre of the screen for 27.5 s, giving a 30-s rest period between contractions, ensuring sufficient time for the post-stimulus response to end. During the rest period, subjects relaxed their hand and refrained from movement. Complete relaxation of the hand was made possible by use of a fingerless glove attached to the grip-force bar; this was worn on the right hand, enabling subjects to release their grip without dropping the bar (Fig. 1A). All stimulus presentation, as well as the recording of outputs from the grip-force bar, was implemented using in-house software written using the Psychophysics Toolbox (Brainard, 1997) in MATLAB (MathWorks, Massachusetts, USA). Subjects were instructed to lie as still as possible and only move the hand needed to perform the task. Only monitoring of movement of the hand and forearm performing the task was carried out.

Within one experimental run, 15 trials of each of the three stimulus durations were presented in a pseudo-random order, providing a total of 45 trials per run. Two runs were acquired per subject, each lasting ~27 min, with a ~15-min break between runs. Before and after each run, subjects attempted to reach two, 3-s-long target profiles of 100%MVF with a 30-s rest period between, akin to that used in a previous study (Fry et al., 2017) to assess fatigue within and between runs. A schematic overview of the experiment is shown in Fig. 1C.

### 2.3 Data collection

Surface Ag/AgCl electrodes (EasyCap GmbH, Germany) to measure electromyography (EMG) were attached to the subject’s right arm, in order to quantify the time at which the subject gripped the bar, as well as to monitor any extra, unwanted movements of the hand during the rest periods. Electrode pairs were positioned in a bipolar configuration over the forearm extensor bundle (channel 1) and forearm flexor bundle (channel 2) muscle groups. EMG data were acquired using an ExG amplifier (Brain Products GmbH, Germany) and BrainVision recorder (v 1.1), with a sampling rate of 1000 Hz and frequency range of 0.016–250 Hz (with 30 dB roll-off at high frequencies). A marker was inserted at the start of the experiment to temporally synchronise with the MEG data.

MEG data were recorded using a 275-channel CTF MEG system (MISL, Coquitlam, BC, Canada) in synthetic 3rd order gradiometer configuration at a sampling rate of 600 Hz. Head localisation coils were attached to the subject at the nasion and preauricular points as fiducial markers. These coils were energised at the start and end of data acquisition to localise the head within the MEG helmet, and to quantify head movement. To coregister brain anatomy with the MEG sensor geometry, a digitised head shape was created using a 3D digitiser (Polhemus, Colchester, VT, USA) relative to the head localisation coils. T_1_-weighted anatomical images were acquired using a 1-mm isotropic MPRAGE sequence on either a 3T or 7T Philips Achieva MR scanner. Coregistration was achieved by matching the digitised head surface with the head surface from the anatomical MRI.

## 3 Data analyses

### 3.1 Pre-processing

#### 3.1.1 EMG

EMG data were downsampled to 600 Hz to match the MEG data sampling rate. An in-house MATLAB programme was developed to determine the exact time of the start and end of the individual hand-grip contractions. For this, EMG data were filtered from 1 to 150 Hz and then rectified. The standard deviation in baseline EMG activity was determined in a time window 13–23 s after the visual cue for contraction offset from all contractions, independently for each EMG channel and subject. This baseline period was used to determine a noise threshold which was defined as three times the standard deviation of the baseline (Cheyne et al., 2008; Muthukumaraswamy, 2010). Subsequently, the onset of contraction was defined as the first time point, in a 0.5-s window either side of the visual cue, when the signal was greater than the noise threshold. If the contraction did not start in this time, the trial was discarded. Similarly, contraction offset was determined as the last time point, in a time window 0.5 s either side of the cue to end contraction, when the signal was greater than the noise threshold. Trials were also discarded if any extra movements occurred during the rest period, detected in both EMG channels.

#### 3.1.2 MEG

MEG data were bandpass filtered from 1 to 150 Hz then visually inspected to remove any trials and channels which contained excessive interference (e.g. due to SQUID resets or excessive muscle activity) using DataEditor (CTF MEG, Canada). This resulted in the removal of, on average, 3 trials out of 15 trials per run, per condition. In addition, markers were added to the MEG data at the times of the contraction start and end, based on the EMG data. The MEG data were then segmented in two ways:

i To investigate the MRBD, the start of an epoch was defined as 3 s before the contraction onset (to ensure all preparatory effects were included).
ii To investigate the PMBR, the data were segmented according to contraction offset. In this case, the start of an epoch was defined as 5 s, 8 s and 13 s before contraction offset. The trials were then segmented into 31, 34 and 39 s epochs (in relation to the cued contraction durations of 2, 5 and 10 s respectively). The epoch lengths were chosen to allow for discrepancies between cued and actual contraction periods.

Following filtering, artefact removal and segmentation, these data were processed using a beamformer spatial filter (see below).

### 3.2 Post-processing

#### 3.2.1 Grip-force and EMG data

Mean grip-force during each contraction was determined, with the first and last 0.5 s excluded so that only steady force output was captured. Force output was calculated as a percentage of the subject’s MVF. The mean rectified EMG signal from each muscle group (forearm extensors and flexors) was determined for each hand-grip contraction (again excluding the first and last 0.5 s of each trial). Separately for the force output and EMG measures, paired Student’s T-tests were used to assess whether any difference in force output/EMG signal occurred in the different runs of the experiment, and a repeated measures ANOVA (RM ANOVA) was used to assess whether there was a systematic difference in force output/EMG signal between durations.

The 100%MVF contractions were analysed to monitor fatigue, with the peak force over a 200-ms epoch used as the measure of MVF during the 100%MVF contractions. This was compared to the subject’s MVF determined at the start of the experiment. Paired Student’s T-tests were used to determine if there were any significant differences in %MVF before and after a run.

#### 3.2.2 Source localisation

Pre-processed MEG data were analysed using a scalar Linearly Constrained Minimum Variance (LCMV) beamformer (Robinson and Vrba, 1998; Van Veen and Buckley, 1988; Van Veen et al., 1997). Pre-processed MEG data were further filtered to the beta band (15–30 Hz), and active and control windows contrasted to determine the spatial signature of task induced beta modulation in the brain. To localise the MRBD, the active window was defined from contraction onset to the cued duration of the contraction (i.e. 2 s, 5 s or 10 s). The control window was defined to start 24 s after contraction onset with a length matching the active window (i.e. terminating at 26 s, 29 s or 34 s). To localise the PMBR, the active window was defined as an 8-s window starting from contraction offset (Fry et al., 2016). The control window was 16–24 s after contraction offset.

The covariance matrices used to compute the weights for the beamformer were created by concatenating the (beta band filtered) data from the active and control windows for the 2-s, 5-s and 10-s trials. Concatenation of data from different task durations was valid as we expect the neuronal sources of the PMBR and MRBD to be the same for all task durations. This concatenation provided the maximum amount of data for the calculation of the covariance matrix thus increasing its accuracy (Brookes et al., 2008). Since evidence suggests the MRBD and PMBR are generated by different sources (Fry et al., 2016; Jurkiewicz et al., 2006), the responses were localised separately using the relevant concatenated active and control window data to calculate two sets of covariance matrices and beamformer weights. Pseudo-t-statistical (Ŧ-stat) images were produced to localise the MRBD and the PMBR by contrasting the relevant active and control windows. A single peak was identified for the MRBD and the PMBR for each subject over all task durations, to ensure source localisation was not biased to any one task duration. The peak of the activity in the left sensorimotor cortex was found for each subject and used to extract time frequency spectrograms (TFSs) at these locations for each subject with maximum signal to noise.

In order to compare the spatial locations of the MRBD and PMBR, the Ŧ-stat maps were transformed from subject space into MNI space using FLIRT (FSL) (Jenkinson et al., 2002; Jenkinson and Smith, 2001). For each subject, MNI coordinates of the peak location of the MRBD and PMBR were recorded. Paired Student’s t-tests were used to separately identify changes in peak locations in the x (left/right), y (anterior/posterior) and z (superior/inferior) direction between the MRBD and PMBR. Group average Ŧ-stat maps were produced for the PMBR and MRBD by averaging across subjects.

#### 3.2.3 Time frequency spectrograms (TFSs)

TFSs were generated with the MEG data filtered into a broader 1–150 Hz band (to capture the broad band response) and all data used to create the covariance matrix. The derived beamformer weights were multiplied by the MEG sensor data to provide estimates of the electrical signal at the identified locations. TFSs were created by frequency filtering these time courses into 31 overlapping frequency bands, with a Hilbert transform used to calculate the envelope of activity within each band. Envelope time courses were averaged over all trials of the same duration (i.e. 2, 5 or 10 s), baseline corrected (baseline was defined as 2–10 s prior to the end of the trial) by subtracting baseline for each band, normalised by dividing by baseline measures (providing a measure of relative amplitude for each subject) and then concatenating in frequency. Resultant TFSs were then averaged over subjects.

#### 3.2.4 Quantification of the beta desynchronization and rebound

In order to quantify the size of the MRBD and PMBR, a curve fitting routine was employed. The beamformer derived time courses were filtered into the beta band (15–30 Hz) and then Hilbert transformed to provide the amplitude envelope of beta oscillations. Amplitudes were baseline corrected and averaged over trials, with the absolute measure of beta amplitude (as distinct from percent change from baseline) maintained. Time courses were then averaged over subjects and the standard error over subjects computed.

A Weibull curve was fitted to the rebound period (Barratt et al., 2017; Liddle et al., 2016), given by

(1) $$\text{f}\left(\text{t}\right)=\text{ ​}\frac{\text{b}}{\text{a}}{\left(\frac{\text{t}}{\text{a}}\right)}^{\text{b}-1}{\text{e}}^{-{\left(\frac{\text{t}}{\text{a}}\right)}^{\text{b}}}\text{,}$$

where a is the scale parameter and b is the shape parameter. A general linear model was used to fit the Weibull curve to the PMBR (defined as when the beta time course amplitude returned to 0 nA m after the MRBD); the scale and shape parameters were iterated to find the best curve fit to the data (minimised sum of squared residuals). These fits were performed for each subject and task duration individually, allowing estimation of the peak PMBR amplitude, time-to-peak, and time the PMBR returned to baseline (defined as when the gradient of the Weibull curve fit was less than 0.0001). Once the best fit to the rebound had been computed, a trapezoid was fitted to the MRBD, using a similar procedure to the PMBR. The time of the vertices of the trapezium were allowed to vary along with the height of the trapezium. The lateral arms of the trapezium were fitted to the downward and upward slopes of the MRBD whilst the base was fitted to the constant MRBD during the movement. Once the best fit was found, the time between the two vertices of the base determined the duration of the MRBD whilst the height of the trapezium determined the amplitude of the MRBD. For each fitted parameter, a RM ANOVA was used to determine if there was a significant effect of stimulus duration on these parameters.

#### 3.2.5 Hidden Markov Model

To gain insight into what is happening to the dynamics of the beta band amplitude envelope at the individual trial level, we used a Hidden Markov Model (HMM) (Baker et al., 2014; Rezek and Roberts, 2005; Woolrich et al., 2013). The brain was parcellated into 78 regions according to the automated anatomical labelling atlas (Tzourio-Mazoyer et al., 2002). Following this, an LCMV beamformer was used to derive a time course of estimated electrophysiological activity for each region (Brookes et al., 2016; Hillebrand et al., 2012). The beamformer was applied with a covariance window encompassing the 1–150 Hz frequency range and a time window capturing the entire experiment. Regularisation was applied to the covariance matrix using the Tikhonov method with a regularisation parameter equal to 5% of the maximum eigenvalue of the unregularised covariance matrix. Timecourses were derived from a single virtual electrode at the centre of mass of each region and symmetrically orthogonalised (Colclough et al., 2015, 2016) for leakage reduction. Prior to application of the HMM the source localised data were downsampled to 100 Hz and frequency filtered to 1–40 Hz (Baker et al., 2014). The Hilbert transform was applied to generate the amplitude envelopes and data were concatenated across subjects and runs.

**Figure S1:**
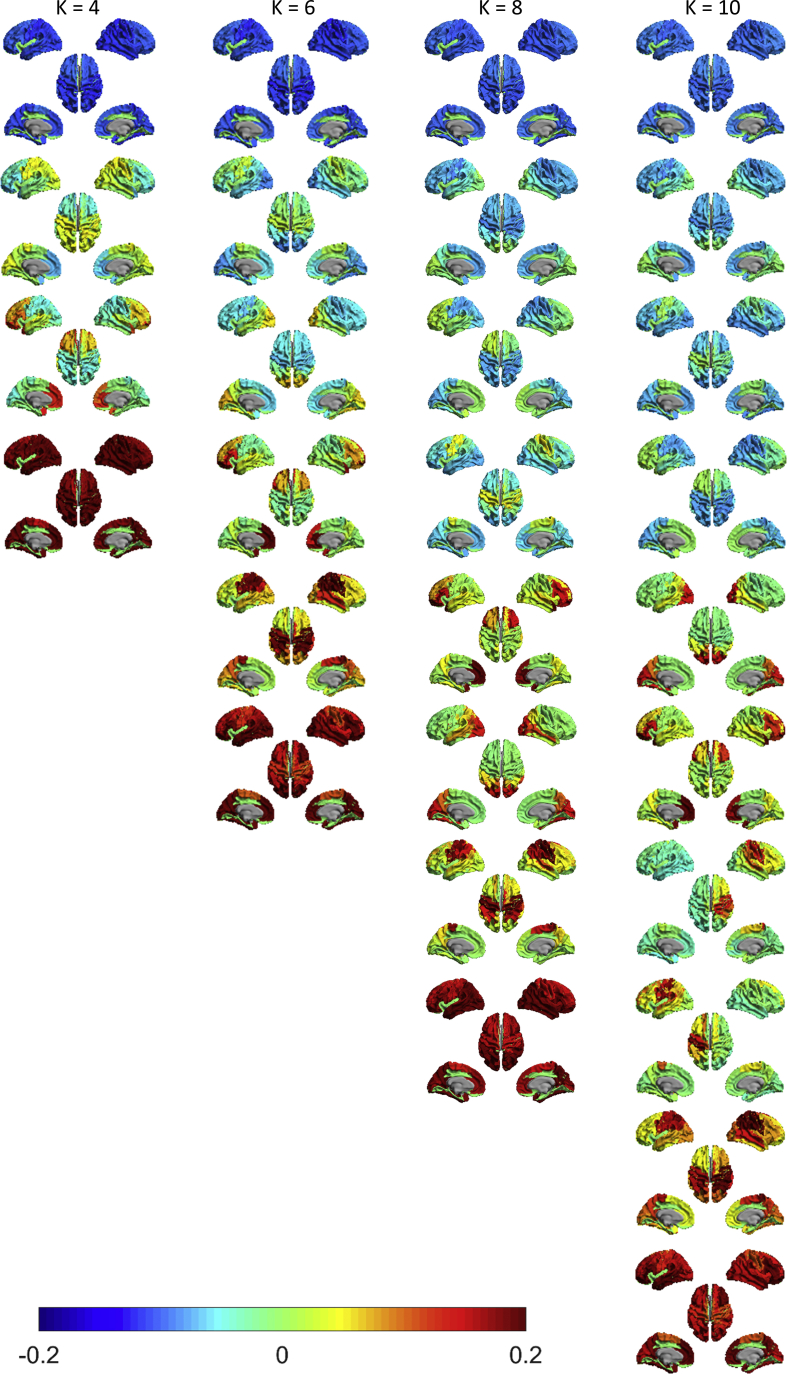


For the HMM itself, analysis is performed on the amplitude envelopes similar to that used in (Baker et al., 2014; Quinn et al., 2018; Woolrich et al., 2013). This assumes that brain activity is well-described by a relatively small number of “states” and that, at any single point in time, only one of these states is active. Note that states are mutually exclusive. In addition, this also assumes that the underlying sequence of states is Markovian; the brain’s current state depends only on its previous state, rather than a complete history of past states. Each state was described by a multivariate normal distribution with a (78 × 1) mean vector and a (78 × 78) covariance matrix. Inference on the HMM is carried out using variational Bayes (VB) (Rezek and Roberts, 2005) to estimate the full posterior distribution on the model parameters (i.e. we obtained a probabilistic description of the likelihood of the unobserved state parameters, and state transition probabilities, conditional on the measured data). In addition, we determined, for every time-point, which of the derived states the brain was most likely in. This was done using Viterbi decoding (Baker et al., 2014; Woolrich et al., 2013). The result is a binary timecourse for each state, showing whether, for any one point in time, that state was the most likely. A HMM with 4, 6, 8 and 10 states was inferred to identify the number of HMM states required to identify a bilateral motor state expected to be modulated by the post-stimulus response as identified in our initial analyses. Each HMM was inferred 10 times to test the variability of the states, and the inference with the lowest free energy was chosen (Quinn et al., 2018). Crucially, the HMM inference was carried out without knowledge of the task timings or structure. Based on this analysis, an 8-state HMM was chosen (see results and Supplementary Figure S1) in agreement with that used previously (Baker et al., 2014).

The resulting 8 binary state timecourses were decomposed to obtain summary statistics. Specifically, for each state and each subject we estimated 1) Fractional occupancy: the fraction of the trial that the brain was in each state. 2) Number of occurrences: number of times a state is visited in a trial. 3) State lifetime: the mean time spent in each state on a single visit. 4) State interval: the mean time between state visits 5) State mean beta amplitude: the mean amplitude of the beta power in the left sensorimotor cortex during state visits (i.e. the timecourse derived from “conventional” analysis above multiplied with the binary state timecourse derived from the HMM to give beta power during state visits). The mean values of these parameters were calculated by averaging over trials and then subjects. Each of these metrics was calculated individually for the three epochs, based on the results from the time-frequency analysis: post-movement period (defined as a 4-s window 1–5 s after movement offset), movement period (defined as a 4-s window −4 to 0 s relative to movement offset) and rest period (20–24 s after movement offset) and averaged over all contraction durations. To test for significant differences in each of the metrics between the three time windows, a RM ANOVA was performed across the subjects and time windows. To interrogate the PMBR period further, the same five metrics were calculated for the three separate contraction durations (2, 5 and 10 s) in the 4-s window after movement offset. A RM ANOVA was then used to test for significant differences of the metrics between contraction durations during the PMBR period.

## 4 Results

From initial assessment of the EMG data, one subject was removed from further analysis due to movement of the hand during the rest periods (EMG data showed hand movement in just under half the trials). Results are therefore reported for the remaining 14 subjects. Following removal of the bad trials, 13 ± 2, 13 ± 2, 12 ± 3 trials out of 15 (average and standard deviation across all subjects and runs) remained for the 2-s, 5-s and 10-s durations respectively.

### 4.1 Grip-force and EMG data

The mean force output (across subjects and durations ± standard deviation) was 29.5 ± 0.8%MVF during run 1 and 29.8 ± 0.6%MVF during run 2, a significant difference (p = 0.02, paired *t*-test). The EMG amplitudes for runs 1 and 2 were 334 ± 175 μV and 306 ± 128 μV respectively on channel 1 (forearm extensor bundle) and 194 ± 75 μV and 181 ± 59 μV, on channel 2 (forearm flexor bundle). These values were not statistically different (p > 0.05, paired *t*-test). The high similarity of the force output and EMG responses across runs, combined with the fact the same number of trials were performed in each run allowed data to be grouped across runs for each subject for the grip-force duration of 2, 5 and 10 s.

**Fig. 2:**
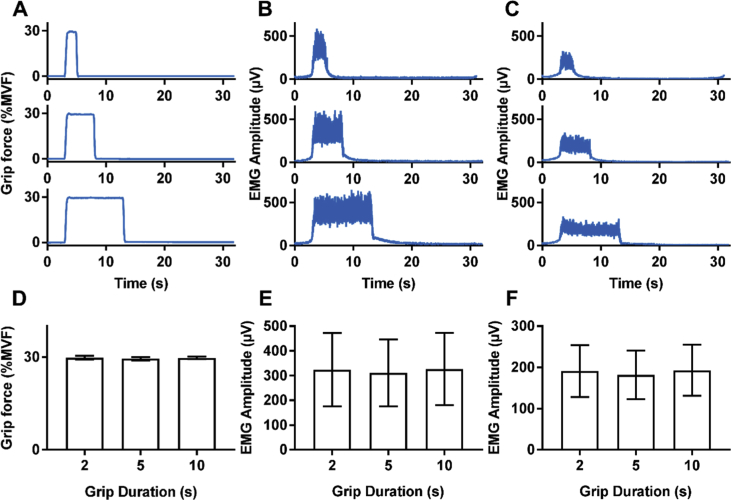
**Behavioural results.** (A–C) Example of output for one subject of (A) grip-force, (B) forearm extensor bundle EMG trace, (C) forearm flexor bundle EMG trace. (D–F) Average 2-s, 5-s and 10-s responses across subjects and runs for (D) grip-force, (E) EMG amplitude in the forearm extensor bundle, (F) EMG amplitude in the forearm flexor bundle.

Single subject time courses of the mean force output and mean EMG responses are shown in Fig. 2A–C. Force data show the high overall performance of the subjects in the task, reaching the 30%MVF and maintaining it for the different durations as required. The EMG traces also indicate neuromuscular activation to perform the task remained the same for the different durations. The mean force output and mean EMG amplitude across all subjects is shown in Fig. 2D–F. A significant difference between the three durations (p = 0.04, RM ANOVA) was found between the force outputs however, this was not seen in the EMG data for either channel. As the differences in mean force output were so small (29.8±0.7%, 29.4±0.7% and 29.5±0.3% for 2, 5 and 10 s grip durations respectively) and no changes in EMG were observed, overall the performance for all three durations was considered to be similar.

The 100%MVFs before and after each run were analysed to assess fatigue during the experiment. Mean force outputs before and after run 1 were 96 ± 12%MVF and 89 ± 12%MVF, respectively whilst they were 87 ± 14%MVF and 83 ± 15%MVF before and after run 2, respectively. Comparing 100%MVF responses no significant differences (paired t-tests, Bonferroni corrected) were seen before and after the task for either run, or when comparing the before or after task 100%MVF measures for each run.

### 4.2 MEG responses

**Fig. 3:**
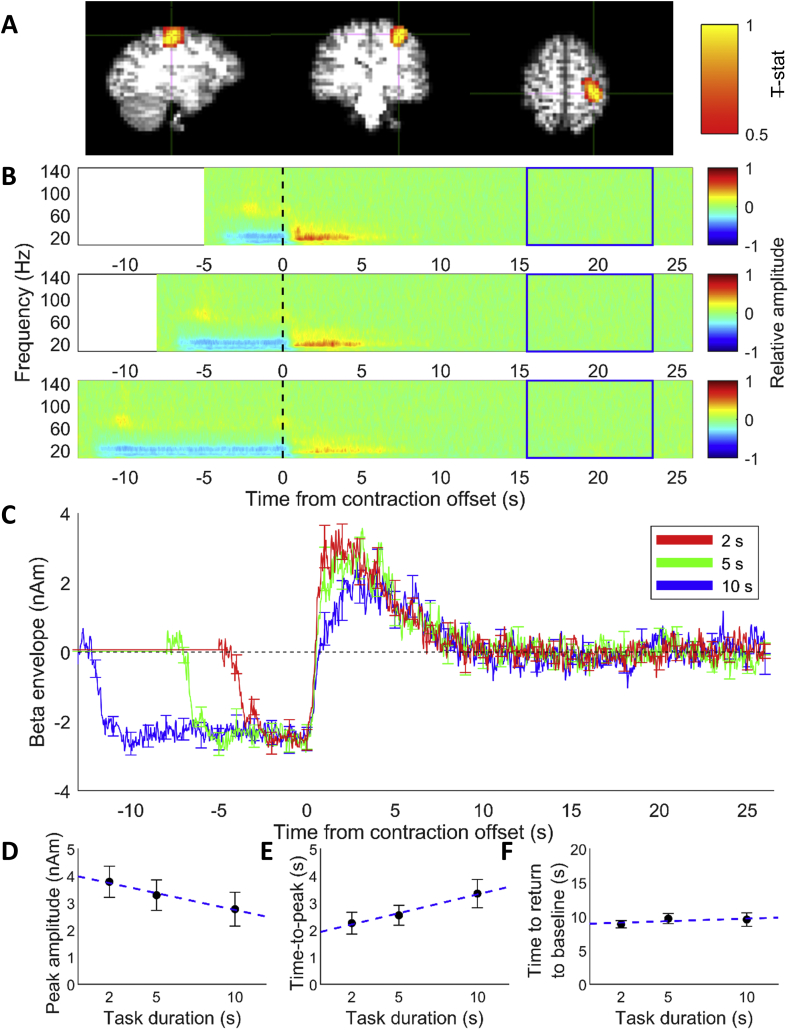
(A) Pseudo-t-statistical map showing the PMBR localised to motor cortex for one run of a single representative subject (radiological view). (B) Average time frequency spectrograms extracted from individual subject PMBR location for the three contraction durations (top panel shows 2-s task duration, middle 5-s task duration and the bottom panel shows 10-s task duration). Spectrograms show the relative change in power for each frequency band where baseline was 16–24 s (blue box). Time zero is cessation of the contraction. (C) Average time courses of beta band (15–30 Hz) amplitude for the three task durations from the peak location of the PMBR across 14 subjects. Responses are aligned to contraction offset (time = 0 s). Red line shows the response to 2-s task duration, green line response to the 5-s task duration and blue line to the 10-s task duration. Error bars show the standard error across subjects. (D–F) Measures from Weibull curves fitted to the PMBR showing effects of task duration. All times reported on y-axes are measured relative to contraction offset. (D) The amplitude of the PMBR peak (R-square 0.98), (E) the time at which peak of PMBR occurs (R-square 0.92) and (F) the time taken for rebound to return to baseline (R-square 0.01). Error bars show the standard error. Blue dashed line shows linear fit of the data.

**Fig. 4:**
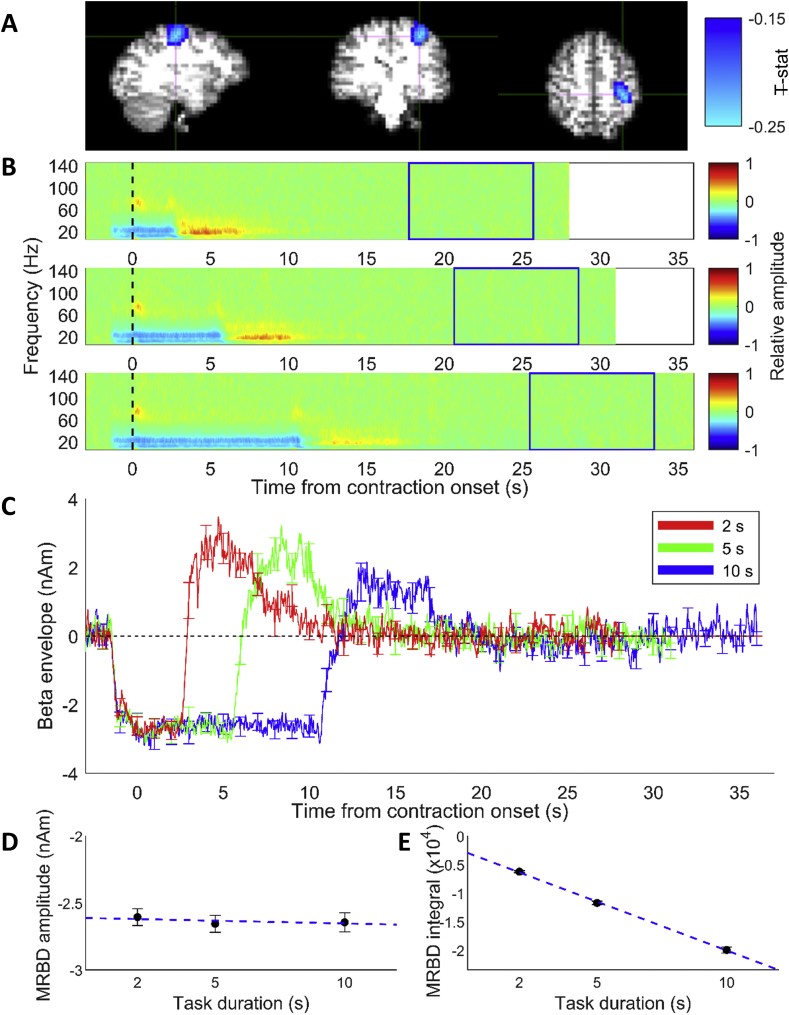
(A) Pseudo-t-statistical map showing MRBD localised to motor cortex for one run of a single representative subject (radiological view). (B) Average time frequency spectrograms extracted from individual subject MRBD location for the three contraction durations. Spectrograms show the relative change in power for each frequency band where baseline was 2–10 s prior to the end of the trial (blue box). Time zero is contraction onset. (C) Average time courses of beta band (15–30 Hz) amplitude for the three task durations from the peak location of the MRBD across 14 subjects. Responses are aligned to contraction onset (time = 0 s). Red line shows the responses to 2-s task duration, green line response to the 5-s task duration and blue line to the 10-s task duration. Error bars show the standard error across subjects. (D–E) Measures from a trapezoid fitted to the MRBD showing effects of task duration. (D) Amplitude of MRBD and (E) integral of MRBD plotted against task duration. Error bars show the standard error across subjects. Blue dashed line shows linear fit of the data.

Contralateral MRBD, localised to the sensorimotor cortex, was found for all subjects, and contralateral PMBR, also localised to sensorimotor cortex, was found in 13 out of 14 subjects. Fig. 3, Fig. 4A show example Ŧ-stat maps for an individual subject for a single run for the PMBR and MRBD, respectively.

Time-frequency spectrograms for the PMBR, averaged across trials, runs and subjects are shown in Fig. 3B, where time zero indicates contraction offset, determined from the EMG trace. As expected, an increase in beta amplitude (i.e. the PMBR) was observed after contraction offset for all three durations, which appears to increase in magnitude as the gripping period decreases (Fig. 3B, red). A slight increase in alpha amplitude was also observed during the PMBR period although this effect was weaker.

Fig. 3C shows the time courses of the beta band amplitude for each task duration averaged over all subjects and runs. Again, it is evident that the PMBR is modulated by task duration, with shorter contractions (red) tending to show higher amplitude compared to longer contractions (blue). Interrogating the PMBR using the Weibull fit showed a significant decrease (p = 0.018, RM ANOVA corrected for multiple comparisons (Benjamini-Hochberg)) in the peak amplitude with increasing contraction duration (Fig. 3D), and a significant increase (p = 0.017, RM ANOVA) in the time-to-peak of the PMBR, (Fig. 3E). No difference (p = 0.55, RM ANOVA) in the time of the PMBR to return to baseline was found between contraction durations (Fig. 3F). The average time to return to baseline was 9 ± 3 s across all subjects and durations. The integral of the PMBR, which combines these effects, showed a significant reduction (p = 0.001, RM ANOVA) with increasing task duration.

Fig. 4 shows results for the MRBD, where time zero represents contraction onset, as determined from the EMG traces. As expected, the TFS revealed a distinct beta (and alpha) band decrease during the grip contraction, with the effect in the alpha band more pronounced than during the rebound period. Furthermore, an increase in gamma band activity (~60–90 Hz) was seen on contraction onset and offset. Fig. 4B and C shows that the MRBD is sustained for the duration of the task, and the MRBD consistently began approximately 2 s before the onset of contraction. The amplitude of the MRBD during the contraction was consistent across task durations, reflected by no significant difference (p = 0.767, RM ANOVA) in MRBD amplitude calculated from the trapezoid fit parameters (Fig. 4D). As expected, the integral of the trapezoid increased linearly with duration (Fig. 4E), reflecting the increase in duration of the MRBD with task duration.

**Fig. 5:**
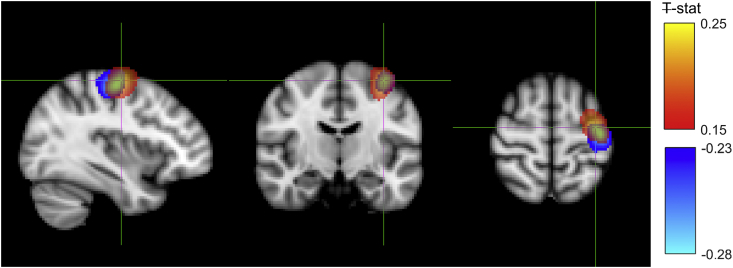
Pseudo-t-statistical map of the group average location of the MRBD (blue, peak (−40, −20, 58) mm) overlaid with the PMBR (red, peak (−36, −12, 62) mm) with the cross hairs at the PMBR peak (radiological view). Ŧ-stat maps were created in individual subject space before normalising to MNI space and averaging over subjects.

Fig. 5 shows the average Ŧ-stat map for the MRBD and PMBR over all subjects, normalised to the MNI brain. The location of the PMBR peak response across all subjects was (−36, −10, 62) mm (MNI coordinates (x, y, z)) while the MRBD peak was at (−40, −20, 58) mm. According to the probabilistic Harvard-Oxford Cortical Structural Atlas (i.e. the fsl “atlasquery” tool) the most likely cortical region relating to the average peak MNI coordinate of the PMBR was precentral gyrus (43%), whilst the peak of the MRBD was split between precentral gyrus (36%) and postcentral gyrus (18%). Whilst there was considerable spatial overlap of the PMBR and MRBD responses, the peak location of the PMBR was significantly more anterior (p < 0.05, paired samples *t*-test) and more medial compared with the MRBD when considered over all subjects.

### 4.3 Hidden Markov Model

**Fig. 6:**
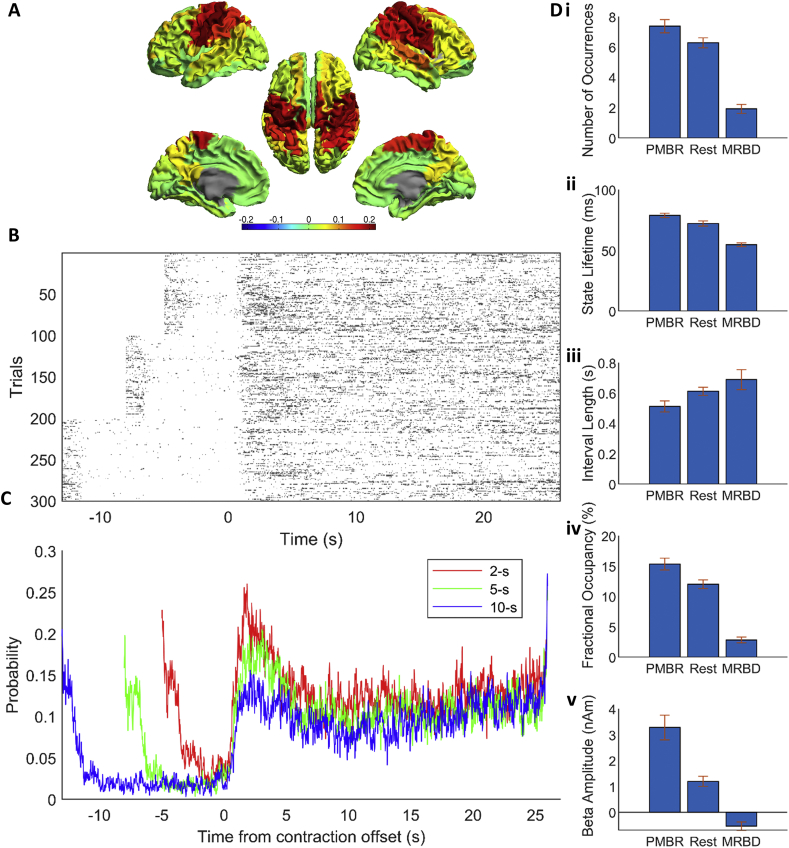
Results from the sensorimotor state of the HMM. (A) State map of state 7 (see Fig. 7) showing a sensorimotor state. (B) Plots of the binary time course for a subset of the first 100 trials for the three conditions (2-s, 5-s and 10-s task duration) against time where dark grey is 1 (in state 7) and white is 0 (not in state 7). (C) Probabilistic time course derived from (B) showing probability of being in state 7 at any given time, for the three conditions. Responses are aligned to contraction offset (time = 0 s), akin to Fig. 3. Red line shows the 2-s task duration, green line is 5-s task duration and blue line is 10-s task duration. (D) Summary metrics for state 7, averaged over all conditions for each subject and then averaged over subjects, separated into three epochs relative to movement offset: post-movement (1–5s), movement (-4 – 0s) and rest (20–24s). Error bars show the standard error over subjects. Additional analyses are shown in Fig. 8.

**Fig. 7:**
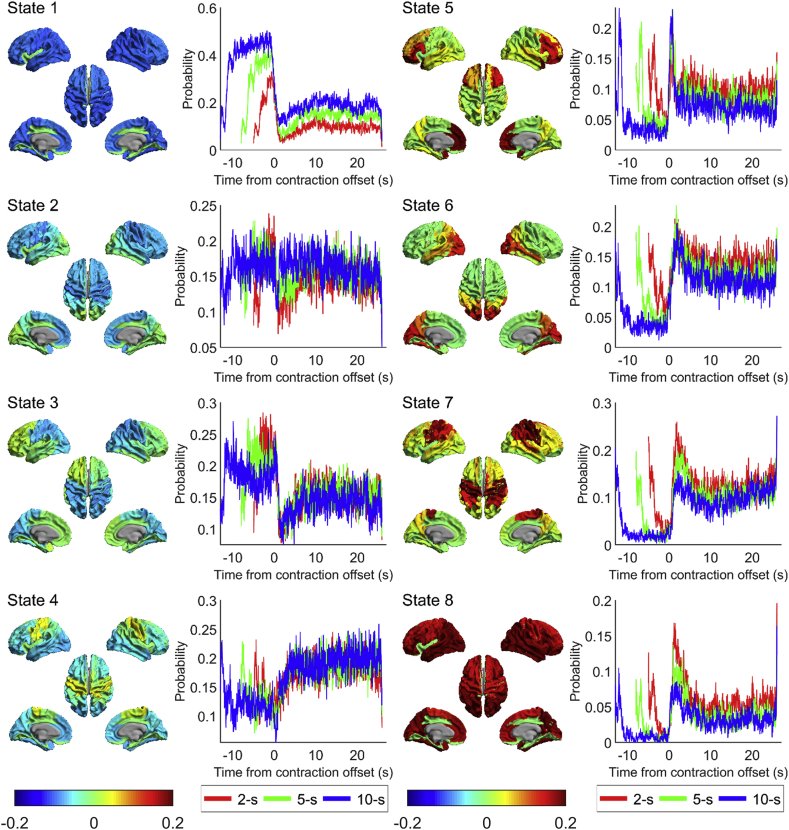
State maps from a HMM inferred with 8 states where red shows brain regions with increased power relative to average and blue decreased power, and associated state probability time courses for the three conditions, where red is 2-s, green is 5-s and blue is 10-s contraction.

The state maps for the HMM with 4, 6, 8 and 10 states are shown in Supplementary Material Fig. S1. When fewer than 8 HMM states were inferred, it was clear that multiple networks were grouped together, implying an insufficient number of states. When 10 states were inferred, the motor network of interest is split across three states which suggests that too many states have been assigned. Therefore an 8-state HMM was used for further analysis in agreement with previous work (Baker et al., 2014). Of these 8 inferred states, a single state was selected for further analysis due to its spatial topography which covered bilateral sensorimotor cortices; results are shown in Fig. 6 (for the results of all eight states, see Fig. 7). Fig. 6A shows the state map (meaning brain areas whose power increased (red) or decreased (blue) (compared to overall average) when the brain entered that state). The spatial topography shows increased power in the sensorimotor network (extending to posterior parietal regions). Fig. 6B shows the binary timecourses of state occurrences for individual trials; these timecourses are shown for a subset of all trials and subjects, with trials on the y-axis and time on the x-axis. Fig. 6C shows a probabilistic interpretation of these data across trials. Note that the sensorimotor state is most likely to be visited immediately after movement offset and least likely to be visited during movement. This means that the probabilistic timecourses mirror the classical MRBD and PMBR (Fig. 3C). Variation with movement duration also mirrors the PMBR results, with a higher probability of the state occurrence for short duration contractions (2s) compared to longer contraction (10s). Given that the HMM was applied (in accordance with (Baker et al., 2014)) in the 1–40 Hz frequency window, the fact that a single state has been derived whose probabilistic dynamics mirrors those of the PMBR, even accounting for parametric variation with stimulus duration, is compelling.

Fig. 6D summarises the state statistics for the sensorimotor network; findings are averaged over conditions and subjects, but calculated separately for three time-windows: during movement, post-movement beta rebound and rest. A significant (p < 0.05, RM ANOVA) difference in number of occurrences (Fig. 6Di), state lifetime (Fig. 6Dii) and fractional occupancy (Fig. 6Div) between all three time-windows was found. This change in fractional occupancy was driven by both a drop in the number of occurrences during the movement period and a change in the length of the state visit. Significant differences between all three time windows were found for the state lifetime, meaning that during the beta rebound, the sensorimotor state was not only more likely to be found, but also its temporal stability was greater (i.e. state visits were longer). No significant differences across the three time windows were found for the mean interval length, i.e. the amount of time between visits to this state (Fig. 6Diii). Interestingly, a significant difference in the beta amplitude in the left sensorimotor cortex when this state was visited was observed between the time windows (Fig. 6Dv), with beta amplitude during state visitations being the greatest during the PMBR. This suggests that the modulation of beta power in the conventional analyses seen in Fig. 3, Fig. 4C is not purely due to the number and duration of visitations to a given state.

**Fig. 8:**
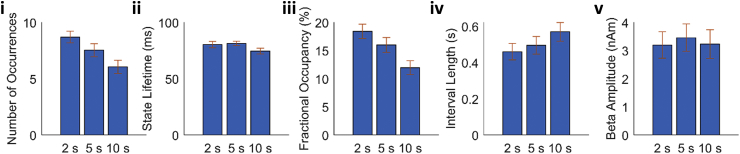
Summary metrics for state 7 (see Fig. 6) during the PMBR period (1–5s post movement offset) for the three contraction durations. Data are averaged over trials within each condition (2s, 5s and 10s contraction duration) and then over subjects. Error bars show the standard error over subjects.

Interrogating the PMBR time window further for different contraction durations, we see a significant (p < 0.05, RM ANOVA) difference in number of occurrences (Fig. 8i) and fractional occupancy (Fig. 8iii) between the three contraction durations. No difference in state lifetime (Fig. 8ii), mean interval length (Fig. 8iv), or beta amplitude during state visits (Fig. 8v) was seen for the contraction durations. This suggests that the modulation in beta power during the PMBR is driven entirely by the number of the visitations to this state, in contrast to the modulation of signals throughout the task time-course. Overall, these results imply that, underlying the beta rebound are rapidly evolving state dynamics which change systematically, not only with movement, but also with stimulus parameters. This will be addressed further in our discussion below.

## 5 Discussion

Using a controlled grip-force task, we have shown that the amplitude of the PMBR is modulated by task duration (for an isometric contraction task), with increased amplitude for shorter contraction durations. This was accompanied by a shorter time-to-peak following contraction offset. We also showed that the total duration of the PMBR was independent of task duration, returning to baseline approximately 9 s after contraction offset. The MRBD was unaffected by task duration. We also showed, in agreement with previous studies, that the MRBD and PMBR localised to spatially neighbouring, but significantly different cortical locations. Finally, we showed that the HMM produces a bilateral sensorimotor network which is most likely to be visited, and with increased temporal stability, during the time window of the PMBR. Moreover, the modulation with contraction duration exhibited by the PMBR was also shown by the probabilistic state dynamics, implying that rapidly evolving network dynamics underlie our observation of systematic variation of the PMBR with stimulus duration. Though the PMBR and MRBD are sustained over several seconds in the TFS responses, the HMM analysis shows that the average motor beta event underlying these effects are only around 100 ms.

Fry et al. (2016) showed that the amplitude of the PMBR, measured from the primary sensorimotor region, decreased and the duration of the response increased with increasing duration of contraction, when rate of force development (RFD) was modulated. This task was relatively complex as both force and duration were simultaneously varied, making it impossible to determine which aspect of the task resulted in the observed changes in PMBR. The authors proposed that it was the duration of the contraction that determined the duration of the PMBR. However here, using a simpler task where only the duration of the contraction was varied, we show that increasing task duration decreases the amplitude of the post-stimulus response and has no effect on PMBR longevity. Nevertheless, the modulation of PMBR amplitude with task duration observed agrees with Fry et al. (2016). Our findings are also supported by those of Heinrichs-Graham et al. (2017) who showed that a longer stimulus duration resulted in a smaller PMBR. However, caution is needed when comparing these studies; in our study the altered PMBRs are observed in the primary sensorimotor cortex, whereas in Heinrichs-Graham et al., PMBRs were reported in higher order brain areas as well as the somatosensory cortex, but not the motor cortex. This difference may be due to differences in task paradigm. In our study, the subjects knew when contraction offset would occur (due to the visual cue), whereas the aim of Heinrichs-Graham et al. was to characterise the effect of not knowing when contraction offset would occur. This may involve recruitment of cognitive networks which potentially gives rise to the spatial differences observed.

The fact that time-to-peak was lower, and amplitude higher for shorter durations suggests that the beta response on contraction offset is a direct response to the duration of the force output, rising more rapidly and to a higher amplitude for shorter task durations (Fig. 3C). We speculate that the most plausible explanation for this finding is linked to task difficulty. Anecdotally, subjects reported finding the task cognitively easier for the longer durations. This was because once the required 30%MVF had been reached and subjects had stabilized their grip it was not difficult to hold that force (as the task had been designed to prevent fatigue). This is supported by quantitative data; there was significantly (p < 0.05, paired Student’s t-test) greater variation in force output recorded across trials for the 2-s task duration (mean over subjects of SD over trials = 1.0 ± 0.5%MVF) than the 10-s task duration (mean over subjects of SD over trials = 0.6 ± 0.3%MVF). Fry et al. (2016) argued that when muscle contraction force is increased, it is conceivable that the perceived task difficultly is increased as it is harder to reach the required force. Indeed their study reports an increase in mean absolute error (a measure of task accuracy) with target force. It is harder to hypothesize how task difficulty is changed by RFD but it is likely that the lower RFD trials were easier as, similar to the longer task durations in the current work, once the desired RFD had been found it could be continued until the end of the prescribed contraction. Again, the reported mean error values supported this suggestion, with smaller errors for lower RFD (Fry et al., 2016). This also agrees with the work from Heinrichs-Graham et al. (2017), which shows reduced PMBR amplitude for slow conditions, which would arguably be easier. Thus in all these cases it appears that the more challenging the task the greater the PMBR. Therefor the PMBR in primary sensory regions may be modulated by top-down feedback mechanisms associated with perceived task difficulty even in these relatively simple tasks.

As summarized in our introduction, beta band responses have been associated with GABAergic inhibition (Cheng et al., 2017; Gaetz et al., 2011; Jensen et al., 2005; Kilavik et al., 2013; Muthukumaraswamy et al., 2013). In support of this, in a previous study, Chen et al. (1999) used transcranial magnetic stimulation (TMS) to explore the functional significance of the PMBR by probing excitability of the motor cortex to drive a muscle twitch in the hand at different lags following median nerve stimulation. They showed maximum cortical inhibition around 200 ms to 1000 ms post stimulus; this timing is closely matched to that of the PMBR. Taking results presented here, it is likely that the peak inhibition is highest and fastest following completion of a task with a shortened duration; or perhaps more generally, peak inhibition is highest and fastest following more challenging motor outputs. We propose that this increased PMBR is a result of increased top-down inhibition required to end the excitatory activity associated with the movement, with greater inhibition required for more cognitively demanding movements.

Interestingly, in the later stages of the response, the rate of decay of the PMBR appears to be the same (from 5 s after movement offset) regardless of task duration and amplitude/latency of the peak of the PMBR (Fig. 3 C and F). It appears that PMBRs of lower amplitude have a wider peak before returning to baseline such that all PMBR responses follow a highly similar trajectory in the later stages of the response, which is surprising. It is tempting to speculate that these later stages are related to fundamental processes such as rebalancing of ionic gradients through after-hyperpolarization currents (Fry et al., 2016; McCormick et al., 1993) which can elicit beta band responses (Kopell et al., 2000; Lu et al., 2004). It is interesting that a similar mechanism of rebalancing of ionic gradients has been proposed as a putative cause of the post-stimulus fMRI response (Lu et al., 2004) and post-stimulus responses across imaging modalities have been linked (Mullinger et al., 2013, 2017). However, if this is the driving mechanism of the later stages of the PMBR it is still challenging to explain why the same trajectory is followed regardless of the peak amplitude of the PMBR, and requires further investigation through modelling and invasive recording approaches.

The HMM analyses provide additional information with regards to the brain regions involved in the PMBR. We have identified a “rebound state” network with the probability of being in this state being the greatest during the PMBR period (Fig. 6C), which is driven by the number of times the state is entered and the length of time spent in the state with each visit (Fig. 6D). The time course of this state has the same characteristics as the beta envelope (Fig. 3C), showing that increasing task duration decreases the probability of being in the state after movement offset (Fig. 6C). The state network identified suggests that it is not only the contralateral sensorimotor region (where our beta band responses were measured from (Fig. 3, Fig. 4)) that exhibits the PMBR, but rather a bilateral network of sensorimotor regions as well as higher order parietal brain regions. These findings therefore support the premise that the PMBR is related to long range integrative processes over distributed networks (Mullinger et al., 2013, 2017; Tewarie et al., 2018), perhaps re-integrating networks which divide during tasks to facilitate unilateral processing (Mullinger et al., 2013, 2017). This finding also complements the theoretical framework of network dynamics (Shenoy et al., 2013) whereby a rapid switching between networks which are recruited during movement preparation and movement onset, are proposed. We observe rapid changes in the number of state visits on movement onset and offset (Fig. 6) whilst the change into resting state from the network primarily recruited in the PMBR period is more gradual. In future work, further insights into the network properties of the PMBR could be obtained using versions of the HMM able to find states that correspond to brain networks with distinct power spectra and phase-locking (Vidaurre et al., 2018a, Vidaurre et al., 2018b).

We also showed from the HMM analyses that the length of each visit to the “rebound” state is greatest in the PMBR period (79±7 ms) and least during the MRBD (55±6 ms) (Fig. 6Dii). Whilst the duration of the visits to the state are longest during the PMBR, they are still an order of magnitude shorter relative to the duration of the PMBR seen through traditional analysis (Fig. 3). The duration of visits into this state are on the time scale of beta bursts (Sherman et al., 2016) perhaps suggesting that this state is denoting beta bursting activity. Bursting activity has been conceptualized as generating the modulations seen in “traditional averaged oscillations” by an increase in likelihood of transient bursts of beta activity at certain phases of a task with no systematic change in the amplitude of the beta bursts across time (Jones, 2016). We observe that the modulation of the PMBR is consistent with this concept, with the frequency of visits to the “rebound” state reducing, whilst the amplitude of the beta band signal remaining constant during the PMBR, with increasing contraction duration (Fig. 8v). This suggests that the modulation of the PMBR seen in Fig. 3 is driven entirely by the number of state visits. In contrast, the amplitude modulation across the task periods i.e. MRBD, PMBR and rest appears from our analyses to be explained by a combination of the bursting hypothesis and the traditional concept of the amplitude, duration and frequency of the beta “bursts” changing across the task. The difference in the apparent underlying sources of the beta envelope modulation (Fig. 3) seen between task periods (MRBD, PMBR and rest) and between contraction durations during the PMBR period suggests the different driving mechanisms generate the different types of modulation. It is plausible that the modulation in bursting activity between task periods is due to a difference in the number of neurons (i.e. size of the network) recruited during these different periods driving different amplitude beta bursts. This would agree with the idea that the MRBD and PMBR are generated through different beta networks, as discussed below.

Our data suggest that the duration of the PMBR is longer than has been reported in recent studies (Gaetz et al., 2010; Heinrichs-Graham et al., 2017; Jurkiewicz et al., 2006; Kilavik et al., 2013; Parkes et al., 2006) and agrees with Fry et al.‘s observation of a long (>6 s) PMBR (Fry et al., 2016). However, it is important to note that the present study, and Fry et al., involved long duration force outputs as distinct from short ballistic (transient) finger movements and so any comparisons should be treated with care. Nevertheless it is possible that the short duration (1–3 s) of the PMBR which has commonly been reported is due to the baseline periods previously used, which typically begin less than 4 s after stimulus/task cessation (Gaetz et al., 2010; Heinrichs-Graham et al., 2017; Jurkiewicz et al., 2006; Kilavik et al., 2013; Parkes et al., 2006). Whilst long inter-stimulus intervals are often used in fMRI paradigm design due to the haemodynamic lag, it has generally been thought unnecessary for electrophysiology recordings. However, these short gaps between stimulus cessation and baseline window will artificially return the time course to baseline giving the impression of a shorter PMBR (and an MRBD that is increased in magnitude). This raises an important methodological point which is further explored in the appendix below.

Finally, as expected from previous work (Fry et al., 2016; Stancak Jr and Pfurtscheller, 1995, 1996), the amplitude of the MRBD remained constant (Fig. 4D) for all task durations and the integral of the MRBD scaled linearly with task duration (Fig. 4E). These findings agree with the previously proposed hypothesis that, during movement, the MRBD acts as a gate which is unaffected by measurable stimulus parameters such as force output (Fry et al., 2016; Stevenson et al., 2011). Fig. 4C shows that the MRBD began at exactly the same time prior to contraction onset, regardless of task duration. The MRBD commenced with the presentation of the visual cue, prior to the contraction. During this preparatory period the MRBD appears to have a slightly lower magnitude than during the contraction itself. This observation is in line with previous work showing that MRBD occurs during movement planning (Kilavik et al., 2013).

## 6 Conclusion

We have shown that, with increasing task duration, the amplitude of the PMBR drops and its time-to-peak increases. There was no effect on overall PMBR duration and no effect on MRBD. Underlying this observation, we have shown that PMBR is likely driven by underlying network dynamics, with a sensorimotor network demonstrating increased temporal stability and increased probability of occurrence during the rebound period. Our work adds weight to the argument that precise control of task parameters enables systematic variation of the PMBR, and hence investigation of its functional role. With increasing evidence of abnormalities of the PMBR in disorders, this will become increasingly important if it is to realise its potential as a biomarker of disease.

## Figures and Tables

**Fig. A1:**
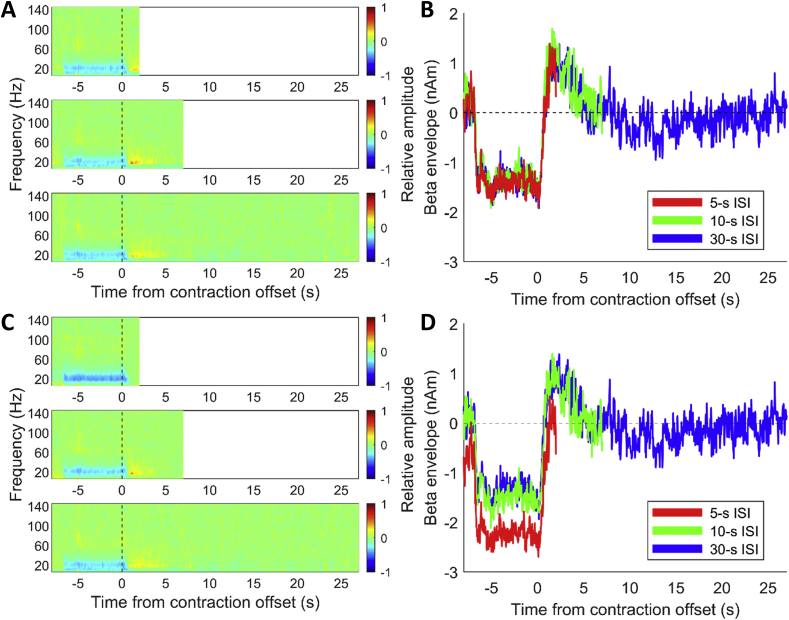
The effect of ISI on the PMBR and MRBD characterisation: (A) Trial averaged time frequency spectra and (B) beta band envelopes for three separate ISIs (5 s (red); 10s (green); 30 s (blue)). Baselines were determined as a single value taken from the long ISI condition. (C&D) Equivalent to (A&B) but where baselines were determined independently from each ISI.

## Appendix 1 timing of the beta rebound

Realising the potential of the PMBR as a biomarker of disease would require its robust characterisation across multiple laboratories. This, in turn, would require standardisation of experimental paradigms. However, to date, the literature is inconsistent regarding how best to illicit a PMBR response. One important question is how long it takes for the PMBR to reach baseline following movement offset. Here, Fig. 3, in agreement with previous work (e.g. Fry et al. (2016)), shows that the PMBR can take up to 10 s to reach a true baseline value following stimulus offset; indeed this is consistent with advice from early studies (Pfurtscheller & Lopes da Silva, 1999) which recommended that a minimum of 10 s is left between trials. However, in recent years this advice is rarely adhered to, with experimenters opting for much shorter inter-stimulus-intervals (ISIs) in order to fit more trials into an experiment. Whilst direct comparisons between the present work and the vast literature on short ISI experiments, which typically employ ballistic finger movements rather than extended force outputs, should be treated with caution, it is possible that such short ISIs, with baselines taken at the end of each trial, could risk underestimation of the magnitude of the PMBR, and overestimation of the MRBD. In this Appendix, we describe a simple experiment to demonstrate this point.

### Methods

Six subjects (3 female, aged 26 ± 1 (mean ± SD) years) took part in a further grip-force experiment in which they were asked to apply force to a bar to match a target profile, as described above in the main text. Here, the duration of force output remained constant (at 5 s) across all trials, but the inter-stimulus interval (ISI) was varied between 5 s, 10 s and 30 s, resulting in a total trial duration of 10 s, 15 s and 35 s for the three different ISIs respectively. The different ISIs were distributed randomly throughout the experiment, with 30 trials for each ISI, which lasted 30 min in total. Data were processed as described above using a scalar beamformer to determine the location in the brain of the maximum beta band change. Both a time frequency spectrum, and the Hilbert envelope of beta band oscillations, were extracted from this location, on a subject-by-subject basis, with results averaged over both trials and subjects. Importantly, we baseline corrected these spectra/envelopes in two ways: 1) The baseline was calculated in the 23 to 27-s window (relative to contraction offset) for the 30-s ISI trials only, and all three conditions (ISIs) were baseline corrected to the same value. 2) Baselines were selected independently for the three trial types, using the 1-2-s window for an ISI of 5 s; the 4-7-s window for an ISI of 10 s and the 23-27-s window for an ISI of 30 s (all relative to contraction offset). These analyses resulted in three time-frequency-spectra and associated beta envelopes (one for each of the three separate ISIs) for the single value baseline correction, and a further three for the independent baseline correction.

### Results and discussion

Fig. A1 shows the 3 time frequency spectra for each of the three ISIs, baseline corrected using a single value derived from the long ISI. Figure A1B shows the corresponding beta envelopes baseline corrected in the same way. Figures A1C and A1D show the same plots, but baseline corrected using baseline values derived from each ISI independently.

The results show that the beta band envelopes follow robust and well characterised profiles regardless of ISI; for the shorter ISIs, the rebound is simply curtailed by the onset of movement. In cases in which the baseline is measured independently for each ISI, it is clear that the “baseline” value is estimated during the PMBR, which leads to an overestimation of the resting beta amplitude, a diminishing PMBR, and an increased (more negative) MRBD. It follows that short ISIs will likely lead to misrepresentation of the MRBD/PMBR and this could, potentially, mask subtle differences in the PMBR between, for example, experimental conditions or clinical populations. An example of this is provided by Rossiter et al. (2014) where differences in MRBD were found between stroke patients and controls, but the ISI for the 3-s grip task was only between 3 and 7s. Thus, it is impossible to determine, with a short ISI, what is driving the differences in the clinical population, especially if MRBD and PMBR do have different neuronal mechanisms. *We therefore recommend that, in future experiments attempting to characterise the PMBR, sufficient time is left between trials to allow a true baseline measure to be derived, in order that robust PMBR characterisation is possible.*
