# Supplementary material for: Post-stimulus beta responses are modulated by task duration
Source: Neuroimage. 2020 Feb 1;206:116288. doi: 10.1016/j.neuroimage.2019.116288 (PMC6985901; doi:10.1016/j.neuroimage.2019.116288)
Supplement: Multimedia component 1 [file mmc1.docx]

**Supplementary Material**

*
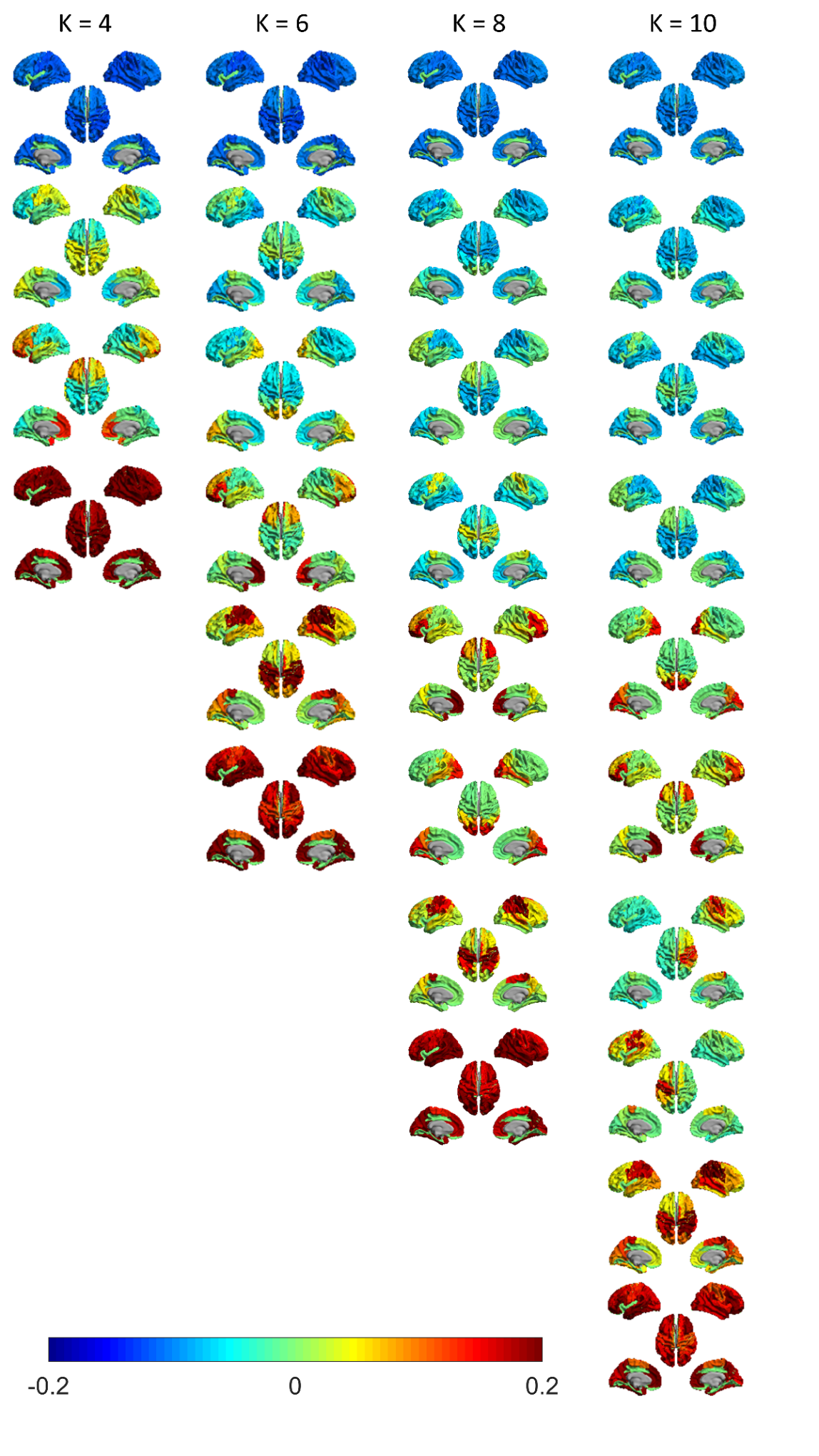
*

***Figure S1.*** *State maps for HMMs inferred with 4, 6, 8 and 10 states where red shows brain regions with increased power relative to average and blue decreased power.*
